# Liver metastases of pancreatic acinar cell carcinoma with marked nuclear atypia and pleomorphism diagnosed by EUS FNA cytology: a case report with emphasis on FNA cytological findings

**DOI:** 10.1186/1742-6413-3-29

**Published:** 2006-12-30

**Authors:** Hong Q Peng, Peter Darwin, John C Papadimitriou, Cinthia B Drachenberg

**Affiliations:** 1 Dept. of Pathology, University of Maryland Medical System. Baltimore, MD, 21201, USA; 2 Dept. of Internal Medicine, University of Maryland Medical System Baltimore, MD, 21201, USA

## Abstract

**Background:**

Acinar cell carcinoma of the pancreas is a rare neoplasm. Although this tumor has been well characterized histologically, the morphological patterns in Fine Needle Aspiration Cytology have not been well defined. Unlike ductal adenocarcinomas, endocrine tumors, and solid pseudopapillary tumors of the pancreas with their characteristic FNA cytological features, acinar cell carcinomas pose a particular diagnostic challenge by sharing many cytomorphologic features with endocrine tumors of the pancreas.

**Case presentation:**

A 37-year-old man presented with lower chest and left upper quadrant abdominal pain. Computed tomography revealed a 7.8 × 7.3 cm irregular, partially cystic mass in the body and tail of the pancreas, and two lesions in the liver compatible with metastases. Subsequently, the patient underwent endoscopic ultrasound-guided fine needle aspiration on one of the two metastatic liver masses.

FNA cytology revealed abundant, loosely cohesive clusters of malignant epithelial cells with vaguely acinar and trabecular formations. The pleomorphic nuclei had fine granular chromatin and occasionally small nucleoli. There were scant to moderate amounts of cytoplasm. Scattered, strikingly large tumor cells with giant nuclei, prominent mitoses and associated necrosis were evident. A pancreatic endocrine tumor was suspected initially, but acinar cell carcinoma of the pancreas was confirmed by immunohistochemistry, cytochemical and ultrastructural studies.

**Conclusion:**

We describe a case of pancreatic acinar cell carcinoma with unusual cytomorphologic features mimicking an endocrine tumor of pancreas, encountered in endoscopic ultrasound-guided fine needle aspiration of a metastatic liver mass and discuss the diagnostic approach for this unusual pancreatic tumor in fine needle aspiration cytology.

## Background

Endoscopic ultrasound (EUS) – guided fine needle aspiration (FNA) is an efficient and safe diagnostic modality that has been increasingly utilized for the primary diagnosis and staging of pancreatic lesions [1-6]. EUS is also a safe and effective method to sample liver lesions. EUS provides access to a significant portion of the liver and to perihepatic structures not readily accessible by a percutaneous approach. It can also detect small focal liver lesions that are not detected on CT scan. Findings of EUS-FNA can establish a cytological diagnosis of suspected liver metastasis and give a definitive M stage that may change clinical management [7-9].

The FNA cytological features of the common pancreatic ductal adenocarcinoma and less common neoplasms such as pancreatic endocrine tumors (PET) and solid psuedopapillary tumor (SPT) are familiar to cytopathologists. However, rare neoplasms like acinar cell carcinomas of the pancreas (PACC), which only account for about 1% of pancreatic exocrine tumors, become a diagnostic challenge when FNA cytology is the sole diagnostic modality. Although PACC has been well characterized histologically [10], the cytological features, especially the morphological patterns of FNA cytology are much less defined.

## Case presentation

**Figure 1:**
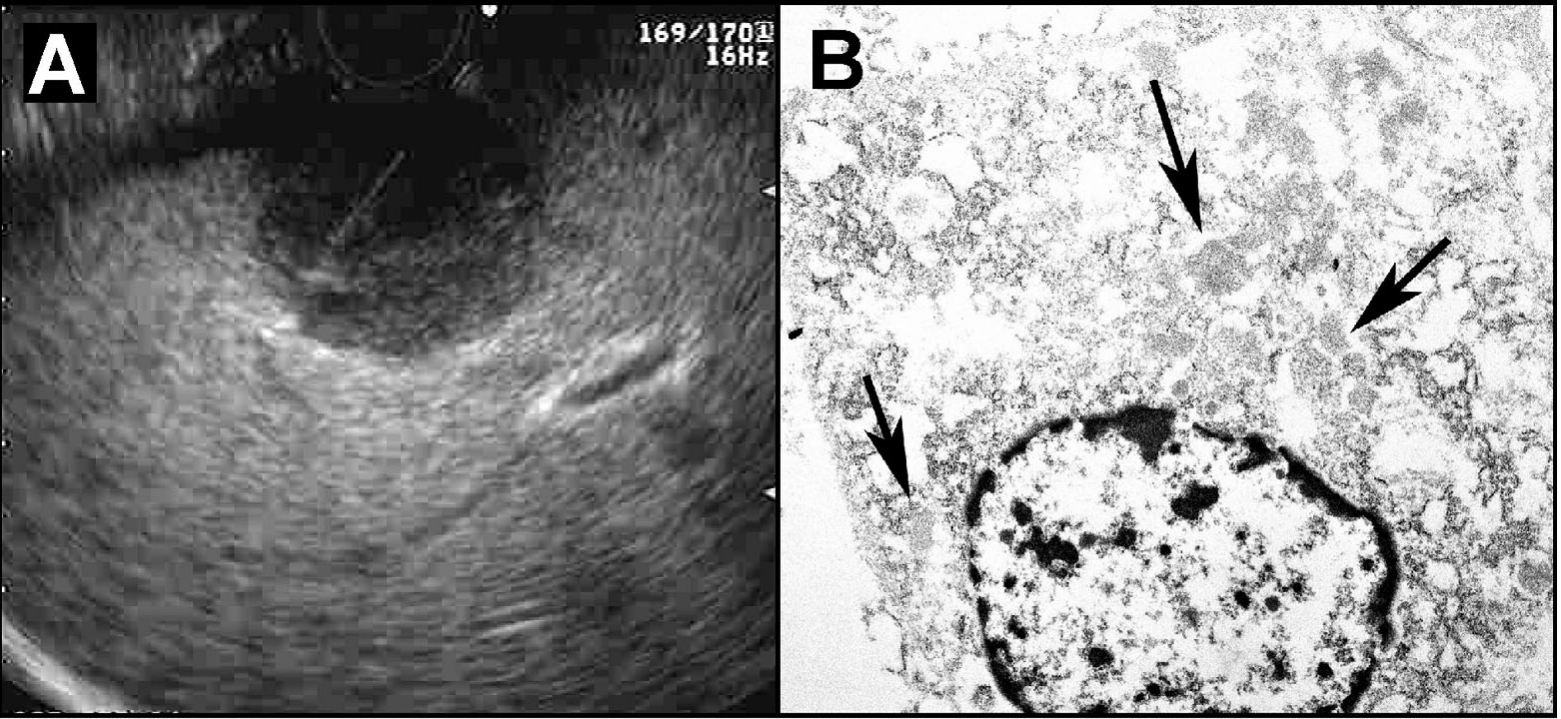
**A. **Endoscopic ultrasound showing the metastatic mass in the left lobe of liver with an aspirating fine needle in it. **B. **Electron micrograph showing clusters of spherical shape and homogenous, medium electron dense zymogen granules in the cytoplasm (arrows).

A 37 years-old Caucasian male with no significant past medical history presented with pain and discomfort in the lower chest and left upper quadrant abdominal area for 6 months. Chest and abdominal computed tomography showed a 7.8 × 7.3 cm irregular, partially cystic mass in the body and tail of the pancreas, suspicious for malignancy. In addition, two lesions were seen in the liver compatible with metastases. He underwent an endoscopic ultrasound (EUS), which revealed a 4.9 × 3.7 cm irregular mass in the pancreatic tail and body. The mass was hypoechoic, heterogeneous, lobulated, multicystic, and septated. There were two masses found in the left lobe and in the perihilar region of the liver, measuring 1.9 × 1.5 cm and 2.2 × 2.2 cm in maximal cross-sectional diameter respectively. Fine needle aspiration was performed on one of the two masses in the left lobe of the liver, after color Doppler imaging was utilized prior to needle puncture in order to confirm a lack of significant vascular structures within the needle path (Figure: 1A). Three passes were made by a 22-gauge needle with a stylet using a transgastric approach. A cytopathologist (HQP) was present and performed a preliminary cytological assessment during the procedure. An adequate sample was obtained after the first two passes but a third pass was requested for special studies due to the unusual morphology seen on site evaluation. The decision to not sample the pancreatic mass was made by the endoscopist.

Two passes were used for 2 sets of Air-Dried smears. One smear of each pass was stained on-site by the Diff-Quick method for immediate assessment. The other Air-Dried smear slides were stained with the Papanicolaou method upon return to the lab for re-hydration. The third pass was directly rinsed in CytoRich Preservative Fluid (TriPath Imaging™, Inc, Burlington, NC 27215) for paraffin-embedded cell block preparation.

## Cytological findings

**Figure 2:**
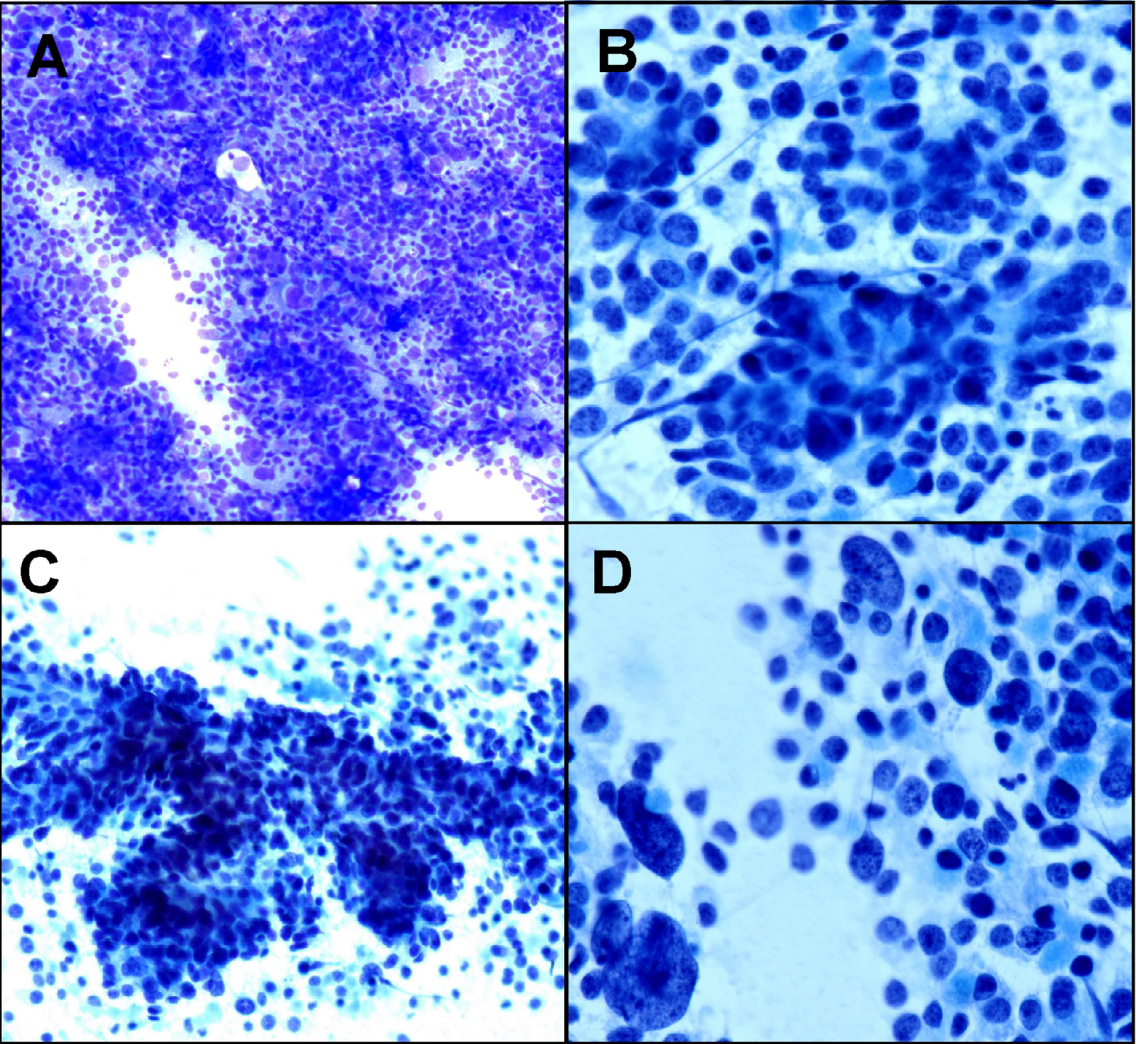
**A. **Hypercellular aspirate with sheet of pleomorphic tumor cells (Diff-Quik, ×10) **B. **Vaguely acinar formation (Papanicolaou stain ×20). **C. **Trabecular pattern (Papanicolaou stain ×10). **D. **Scattered large tumor cells with giant nuclei (Papanicolaou stain ×20).

Both the Diff-Quick and Pap stained smears yielded a hypercellular aspirate containing abundant malignant epithelial cells arranged singly, in sheets and loosely cohesive clusters with vaguely acinar and trabecular formations. The neoplastic cells had pleomorphic nuclei varying from round or oval to carrot shaped, fine granular chromatin, and occasionally small or prominent nucleoli. The tumor cells had scant to moderate amounts of cytoplasm. In addition, scattered large tumor cells with giant nuclei were obvious (Figure: 2). Rare single benign hepatocytes were present in the background.

**Figure 3:**
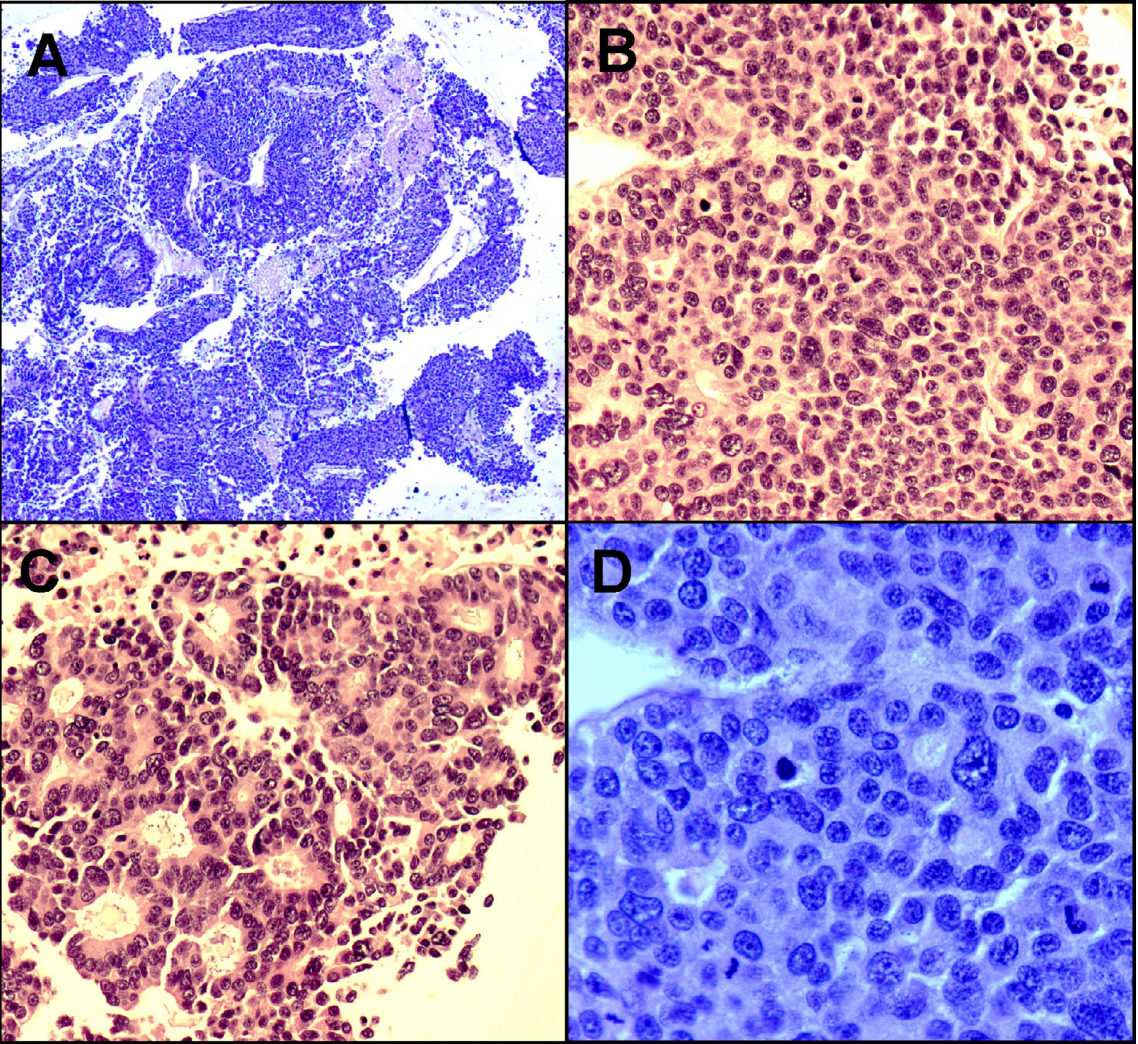
**A. **On cell block, the tumor cells arranged in thick trabeculae, poorly formed glands or acini. Necrosis is evident. (H&E stain ×4); **B. **Solid pattern. (H&E stain ×20); **C. **Acinar pattern with uniform, eccentric located nuclei with abundant eosinophilic cytoplasm. (H&E stain ×20); **D. **Tumor cells with pleomorphic, centrally located nuclei, small to prominent nucleoli and scant to moderate amount of cytoplasm. Brisk and abnormal mitosis are evident. (H&E stain ×40)

On the H&E stained slides from the cell block, the tumor cells arranged in solid sheets, thick trabeculae, and poorly formed glandular or acinar patterns. Most tumor cells had scant to moderate amounts of eosinophilic and granular cytoplasm, relatively uniform and centrally located nuclei, and small or conspicuous nucleoli. Scattered giant tumor cells with bizarre, pleomorphic nuclei, brisk and abnormal mitoses as well as associated necrosis were evident (Figure: 3). A metastatic PET was suspected initially.

## Ancillary studies

**Figure 4:**
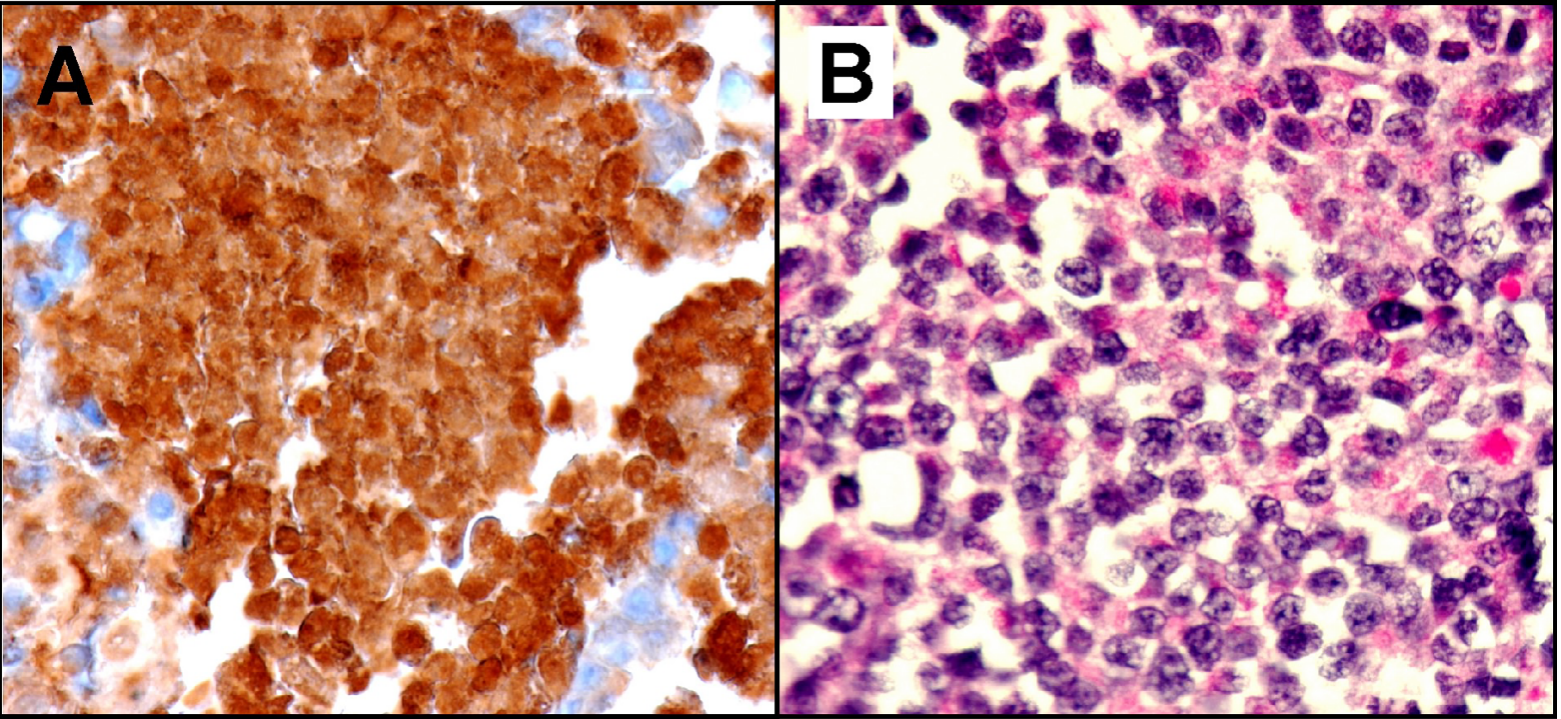
**A. **The tumor cells are strongly positive for alpha-antitrypsin (immunoperoxidase stain ×40) **B. **Tumor cells with positive cytoplasmic PAS-D stain. (Periodic Acid-Schif with diastase stain ×40)

Immunohistochemistry studies on the cell block showed intense cytoplasmic staining for pancytokeratin and α-1-antitrypsin (Figure: 4-A), focal staining for chymotrypsin and cytokeratin7, and negative staining for chromogranin, synaptophysin, CD56, NSE, trypsin, vimentin, cytokeratin20 and BerEP4 on the tumor cells. The tumor was positive for Periodic Acid-Schiff with diastase by cytochemical study (Figure: 4-B).

Electron microscopy was performed in deparaffinized tissue obtained from the cell block. On ultrastructural evaluation the tumor cells were polygonal with moderately abundant cytoplasm. Despite the deparaffinization artifacts, zymogen granules were easily identified. The granules were scattered in the cytoplasm with a roughly spherical shape and homogenous, medium electron dense contents (Figure: 1B). There were no neuroendocrine type granules.

## Discussion

The rarity of PACC has resulted in limited details in the literature regarding its cytomorphology. Before Labate, et al published the cytologic features of acinar cell carcinoma in the largest case series of 7 cases[11], there were only two single case reports [12,13] and one paper with 2 cases [14]. Recently, Stelow et al reviewed FNA cytological features of four cases of PACC encountered in a 5-year period in two hospitals [15]. The typical cytological features described by these authors include isolated and loose clusters of uniform cells with smoothly contoured, eccentric nuclei and clumped chromatin containing one or two conspicuous nucleoli. The cells are small to moderate-sized and polygonal with a moderate amount of granular cytoplasm. Although these features are noted for PACCs, many of them can be shared with PET, which can show rosette formation and even acinar formation with cells that have both granular cytoplasm and clumped chromatin with prominent nucleoli [16]. However, PET tends to display prominent nuclear atypia and pleomorphism, which has never been described in PACC [11-15]. In Stelow's case series, two of the four cases had been misdiagnosed as PET in the final cytological diagnosis, whereas one case was correctly diagnosed as PACC based on cytomorphology [15]. Our case presents many features of the shared cytomorphology between PACC and PET with a striking nuclear pleomorphism and atypia, which led to us to suspect PET initially. Although metastatic ductal adenocarcinoma, SPT, and pancreatoblastoma should be considered in the differential diagnosis, the lack of cytomorphological features for ductal adenocarcinoma and SPT and the absence of squamous corpuscles and/or heterologous elements for pancreatoblastoma excluded them from our differential diagnosis. After the first panel of immunomarkers failed to show any neuroendocrine differentiation, we extended our differential work-up to include acinar cell carcinoma and hepatocellular carcinoma, although the latter is less likely. Subsequently, electron microscopy, immunochemistry and cytochemical studies confirmed the diagnosis of PACC.

Histologically, PACC has been well characterized as having acinar, solid, trabecular and glandular patterns of growth [10]. The acinar pattern consists of cells growing in well-formed acini with basally oriented nuclei and relatively abundant eosinophilic cytoplasm. The solid pattern is characterized by sheets and cords of cells exhibiting minimal cytoplasm and lacking the characteristic eosinophilic granular appearance. The nuclei are usually centrally located. The glandular and trabecular patterns are less frequently encountered and are always accompanied by at least one of the major patterns either acinar or solid. A gyriform arrangement is occasionally seen. The cytological features described in the literature are mostly derived from the predominantly acinar pattern or mixed acinar and solid pattern. Neither the cytological features of other patterns nor the nuclear pleomorphism in PACC have been described before. Only Klimstra et al. mentioned in their histological study that rarely there are pleomorphic nuclei present in PACC, and these are invariably located in the solid areas [10]. Our case uniquely shows a variety of cytological features of a predominantly solid pattern combined with trabecular and acinar patterns, as well as unusually striking nuclear pleomorphism and atypia encountered in a liver metastasis of PACC. Unfortunately, we do not have cytological or histological morphology from the primary site for comparison due to the advanced stage of the patient. A search through the English-language literature revealed only two reports of PACC diagnosed based on a metastatic site. In Labate's case series, seven histologically confirmed acinar cell carcinoma patients had cytologic material from liver, pancreas, or peripancreatic lymph node. The authors did not mention how many patients from each source. However, only two of these patients were correctly diagnosed on cytology, three were originally diagnosed as islet cell tumor, one was "suspicious for adenocarcinoma," and one was "positive for neuroendocrine carcinoma" [11]. The other case report of a PACC diagnosed on a metastatic tumor  extrinsic to the common bile duct was based on histology and it was initially diagnosed as a well differentiated adenocarcinoma of pancreas  by light microscopy but confirmed as PACC by electron microscopy [17]. These further confirm the difficulty of diagnosis of acinar cell carcinoma based on cytology alone.

One of the unique features associated with EUS FNA is the presence of a variable number of normal GI epithelial cells and other benign cells from the target organs. While essential to making the correct diagnosis, distinguishing normal from abnormal cellular elements is not always easy, especially when dealing with a well differentiated adenocarcinoma in the pancreas and a well differentiated hepatocellular carcinoma in the liver [22]. However, it was not an issue in our case since we had a highly cellular specimen containing almost exclusively markedly atypical and pleomorphic neoplastic cells with only a few normal hepatocytes in the background.

## Conclusion

A rare neoplasm like PACC often becomes challenging diagnostically when FNA cytology is the sole diagnostic modality. Due to many morphologic similarities at the microscopic level between PACC and PET, ancillary studies such as immunohistochemistry, cytochemistry and electron microscopy are often needed. The prognosis and treatment options for PACC and PET are different especially when resection is not an option, thus making it very important to differentiate between the two. Many studies have shown that regardless of the imaging modality used, the presence of a pathologist on site improves sampling and sensitivity by ensuring adequate tissue collection and sample preparation [18-21], and our present case supports this conclusion. When we encountered an unusual pancreatic tumor during the on-site evaluation, we ensured an additional pass for the cell block preparation necessary for ancillary studies, an essential element in making the final definitive diagnosis based only on FNA cytology.

## Competing interests

The author(s) declare that they have no competing interests.
